# Genome wide DNA methylation landscape reveals glioblastoma’s influence on epigenetic changes in tumor infiltrating CD4+ T cells

**DOI:** 10.18632/oncotarget.27955

**Published:** 2021-05-11

**Authors:** Marpe Bam, Sreenivasulu Chintala, Kaleigh Fetcko, Brooke Carmen Williamsen, Seema Siraj, Sheng Liu, Jun Wan, Xiaoling Xuei, Yunlong Liu, Adam T. Leibold, Mahua Dey

**Affiliations:** ^1^Department of Neurological Surgery, School of Medicine and Public Health, University of Wisconsin-Madison, Madison, WI, USA; ^2^Department of Neurosurgery, Indiana University School of Medicine, Indianapolis, IN, USA; ^3^Department of Bioinformatics, Indiana University School of Medicine, Indianapolis, IN, USA; ^*^These authors contributed equally to this work

**Keywords:** glioblastoma, malignant glioma, CD4+ T cell, DNA methylation, brain cancer

## Abstract

CD4+ helper T (Th) cells play a critical role in shaping anti-tumor immunity by virtue of their ability to differentiate into multiple lineages in response to environmental cues. Various CD4+ lineages can orchestrate a broad range of effector activities during the initiation, expansion, and memory phase of endogenous anti-tumor immune response. In this clinical corelative study, we found that Glioblastoma (GBM) induces multi- and mixed-lineage immune response in the tumor microenvironment. Whole-genome bisulfite sequencing of tumor infiltrating and blood CD4+ T-cell from GBM patients showed 13571 differentially methylated regions and a distinct methylation pattern of methylation of tumor infiltrating CD4+ T-cells with significant inter-patient variability. The methylation changes also resulted in transcriptomic changes with 341 differentially expressed genes in CD4+ tumor infiltrating T-cells compared to blood. Analysis of specific genes involved in CD4+ differentiation and function revealed differential methylation status of TBX21, GATA3, RORC, FOXP3, IL10 and IFNG in tumor CD4+ T-cells. Analysis of lineage specific genes revealed differential methylation and gene expression in tumor CD4+ T-cells. Interestingly, we observed dysregulation of several ligands of T cell function genes in GBM tissue corresponding to the T-cell receptors that were dysregulated in tumor infiltrating CD4+ T-cells. Our results suggest that GBM might induce epigenetic alterations in tumor infiltrating CD4+ T-cells there by influencing anti-tumor immune response by manipulating differentiation and function of tumor infiltrating CD4+ T-cells. Thus, further research is warranted to understand the role of tumor induced epigenetic modification of tumor infiltrating T-cells to develop effective anti-GBM immunotherapy.

## INTRODUCTION

Naïve CD4+ helper T cell population is known for its polyfunctionality and highly plastic characteristics [1, 2]. To mount an effective immune response naïve CD4+ T-cells are capable of differentiating into specific subpopulations with distinct effector functions such as Th1, Th2, Th17, Treg etc. [3]. These CD4+ T cell subsets play a crucial role in modulating immune response in variety of condition such as infection, allergy, autoimmunity and cancer [4]. It is becoming increasingly evident that in the context of anti-tumor immune response CD4+ T-cells are essential for the generation and maintenance of effective anti-tumor CD8+ cytotoxic T-cell response [5]. Th1 polarized CD4+ T-cells orchestrate and maintain anti-tumor immune response by directly secreting effector cytokine such as IFNγ and TNFα and also by activating and supporting cytotoxic CD8+ T-cells [6, 7].

Upon stimulation by antigen presenting cell, expression of key transcription factors results in naïve CD4+ T cell polarization and differentiation into specific lineage. T-bet, GATA3, FOXP3 and RORγt expression determines Th1, Th2, Treg and Th17 cell fate commitment, respectively [8–10]. In addition, it has been shown that CD4+ T cells have functional plasticity, where they contain all of the necessary machinery to behave like other T cell lineages with the proper impetus [1]. Recent studies have shown that epigenetic changes in DNA methylation is involved in CD4+ T-cell polarization resulting in differential Th1 and Th2 cytokine secretion [11, 12]. In the tumor microenvironment (TME), lineage commitments of CD4+ T cells reflect initiation of new programs of gene expression within tumor infiltrating naïve T cells [13]. These gene expression changes in tumor infiltrating CD4+ T-cells may be mediated by epigenetic events such as DNA methylation.

Glioblastoma, a neoplasm of glial origin, is the most common primary brain tumor in adults and accounts for 52% of all gliomas [14]. It is the most aggressive brain tumor with very poor prognosis and 100% recurrence rates. The GBM tumor microenvironment is known to be extremely immunosuppressive, possessing multiple unique properties including i) impaired cellular immunity [15–17] no dearth of tumor infiltrating T cells [18] iii) high levels of TGFβ secreted by resident as well as circulating microglia [19] and iv) expression of several inhibitory ligands, eliciting anergy and apoptosis of cytotoxic lymphocytes in the TME, immune checkpoints expression, and increased infiltration of immunosuppressive cells [20–23]. It has been reported that the GBM TME influences the CD4+ TIL’s plasticity, which dictates whether they possess immunotolerant or anti-tumor activity [24]. However, the type of endogenous immune response to GBM and molecular mechanism that regulates tumor infiltrating CD4+ T-cell lineage in the TME is not known.

In this clinical corelative study we show that GBM microenvironment lacks directed anti-tumor effector phenotype of tumor infiltrating CD4+ helper T-cells, instead it is characterized by mixed effector phenotypes of multiple lineages. Genome wide methylation sequencing showed 13571 uniquely differentially methylated regions (DMR), mostly concentrated around the TSS, in the CD4+ T cells from GBM patient tumor compared to blood. In particular, we observed differential methylation in the lineage specific genes TBX21, RORC, GATA3, FOXP3 and key cytokine genes IL10 involved in the development and function of specific subpopulations of CD4+ T cells. Furthermore, combining transcriptomic data from RNAseq analysis with DNA methylation, we observed differential methylation of gene sets specific for CD4+ T cells including Th1, Th2, Th17 and iTregs in GBM tumors, although with significant interpatient variability. These genes had DNA methylation patterns corroborating well with their RNA expression patterns indicating possible regulation by DNA methylation. Moreover, the RNAseq analysis revealed 341 significantly dysregulated genes in tumor associated CD4+ T cells compared to blood. Additionally, we observed differentially expressed ligands specific to several CD4+ T cell receptors on the GBM cells. In conclusion, our data for the first time, report unique DNA methylation pattern and gene expression profiles in GBM associated tumor infiltrating CD4+ T cells compared to CD4+ T-cell from the blood of the same patient and some of their ligands on the GBM cells suggesting that CD4+ T cells function and differentiation may be influenced by the GBM TME by way of epigenetic mechanisms such as, DNA methylation. These corelative findings need to be further validated in future studies to optimize immunotherapy for GBM patients.

## RESULTS

### Identification of differentially methylated regions

**Figure 1:**
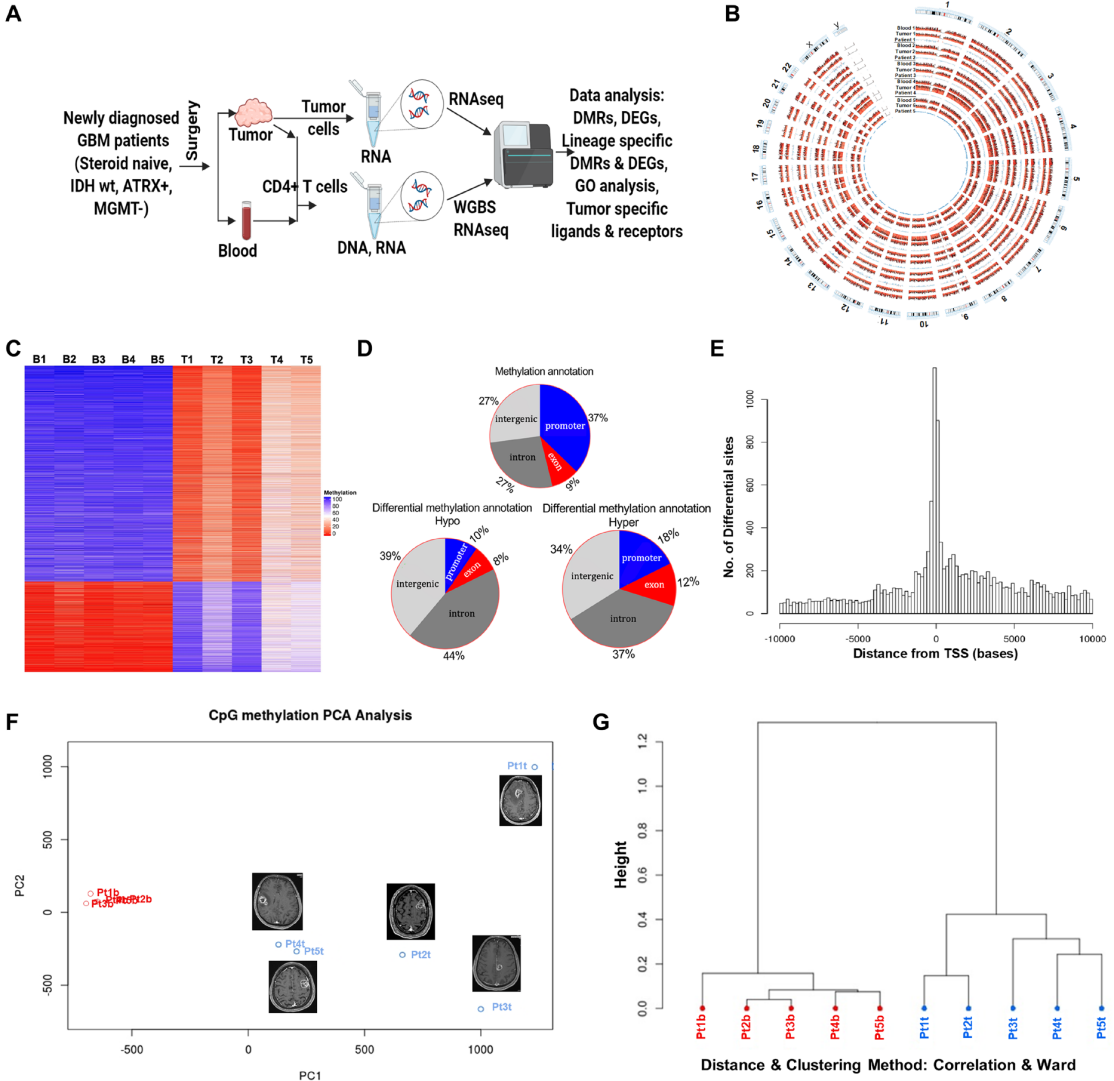
DNA methylome analysis of tumor infiltrating and peripheral blood CD4+ T cells from GBM Patients. (**A**) Work flow of the proposed study. (**B**) Circos plot showing whole genome bisulfite sequencing methylation data for chromosome 1-22 and sex chromosome X and Y for matching tumor infiltrating and blood CD4+ T-cells for all five patients. (**C**) Heatmap representing DMRs in all the samples as both hyper (blue) and hypomethylated (red) in blood and tumor CD4+ T cells from GBM patients (B and T in the sample names, on top of the heatmap, stands for blood and tumor, respectively. The numbers indicate the sample number assigned by us). Scale: red (0) represents hypomethylation and blue (100) indicates hypermethylation. (**D**) Stratification of DMRs into hypo and hypermethylated segments representing the promoter, exon, intergenic or intragenic regions on the basis of their genomic location indicating frequency of DMRs per location. (**E**) Average distance of DMR’s (shown in the horizontal axis) from the transcription start site (TSS) of the closest gene. (**F**) Principal component analysis (PCA) of DMR methylation in tumor and blood CD4+ T cells. MRI of the brain with contrast demonstrating the location and size of the tumor in all 5 patients. (**G**) Unsupervised hierarchical clustering dendrogram of DMRs in CD4+ T cells from tumor and blood (red; patient blood and blue; patient tumor).

We isolated CD4+ T-cells from the tumor infiltrating lymphocytes (TIL’s) and peripheral blood of GBM patients and found increased CD4+ T-cells from multiple lineages (Th1, Th17 and Treg) in the TIL’s compared to blood (Supplementary Figure 1A). To investigate the role of tumor microenvironment (TME) in regulating CD4+ T-cell lineages, we performed whole-genome bisulfite sequencing (WGBS) to decipher the DNA methylome of CD4+ T cells isolated from tumor and blood from 5 newly diagnosed GBM patients (Figure 1A and 1B, Table 1). Using matched samples from five different patients we obtained robust data from all samples with about 40M total reads per sample and an average 31-fold coverage for each CpG per population (Supplementary Figure 1B–1D). Using strict definition of at least 60% difference in DNA methylation, we observed significant difference in the methylation pattern between GBM infiltrating CD4+ T-cells compared to the blood with ~75% of the DMRs were hypomethylated and ~25% were hypermethylated in tumor CD4+ T cells (Figure 1C). Supplementary Table 1 list the top 10 significant DMRs and their associated genes in tumor CD4+ T cells compared to blood CD4+ T cells. In addition, we found that the methylation pattern of the blood CD4+ T cells between patients is very similar, however there is considerable differences in the methylation pattern between patients in GBM infiltrating CD4+ T cells. There were 13571 unique differentially methylated regions (DMR) and, annotation study of the tumor CD4+ T cells revealed that majority of DMRs are located in the promoter region (37%) followed by introns and intergenic regions (27% each), and exons (9%). Within the hypomethylated DMRs, majority of alterations occurred in intron and intergenic regions (44% and 39% respectively) as compared to promoter (10%) and exon (8%) regions. Whereas, in the hypermethylated DMRs, the intronic and intergenic regions were 37% and 34% respectively, the promoter region was 18% and exonic regions was 12% (Figure 1D). We observed concentration of DMRs within ~2 Kb upstream and downstream of TSS (Figure 1E). Principal Component Analysis (PCA) and Hierarchical Cluster Analysis (HCA) of CpG methylation showed distinct variability between blood and tumor CD4+ T cells of GBM patients with no significant interpersonal variability in the CD4+ T cells from the blood but significant individual variability in the CD4+ T cells from the tumor (Figure 1F and 1G, respectively). These results indicated that TME has a profound effect on the methylome of GBM infiltrating CD4+ T cells and they have a distinct methylation pattern compared to the blood CD4+ T-cell. In addition, there is significant inter-patient variability in GBM infiltrating CD4+ T-cell methylation pattern.

**Table 1:** Clinical characteristics of patients included for the sequencing analyses

Patient	Age	Gender	Pathology	Molecular stratification	Location	Date of diagnosis	Date of death
Pt 8	57	M	GBM	IDH wt, ATRX pos, MGMT not assessed	Right frontal	3/24/2017	4/3/2018
Pt 9	70	M	GBM	IDH wt, ATRX pos, MGMT un-methylated	Left frontal	3/30/2017	1/4/2018
Pt 10	59	M	GBM	IDH wt, ATRX pos, MGMT methylated (intermediate)	Left frontal	3/31/2017	alive
Pt 12	61	F	GBM	IDH wt, ATRX pos, MGMT not assessed	Right Parietal	7/7/2017	alive
Pt 13	70	F	GBM	IDH wt, ATRX pos, MGMT not assessed	Left frontal	7/10/2017	12/24/2017

### RNA expression profile validates DNA methylation pattern

**Figure 2:**
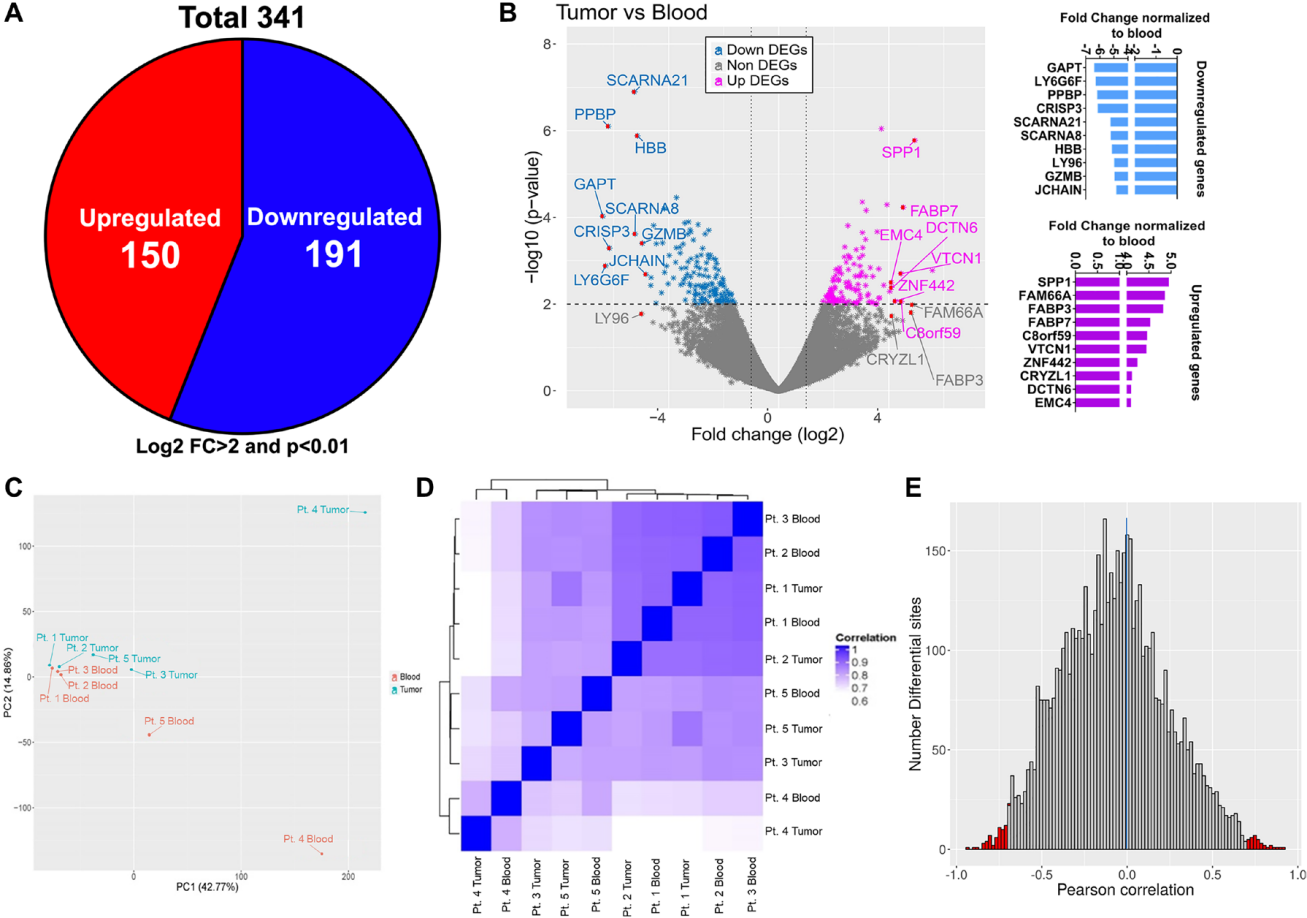
Transcriptome profile of matched tumor and blood CD4+ T cells and correlation with methylation dataset. (**A**) Total number of dysregulated genes (both up and down regulated; numbers shown in the pie chart) in tumor associated CD4+ T cells compared to blood CD4+ T cells. Only genes having log2 fold change > 2 and *p* value < 0.01 were included. (**B**) Volcano plot showing all the differentially expressed genes (both down- light blue and upregulated- pink). Top 10 dysregulated genes are marked with arrows in the volcano plot and their fold change values normalized to blood is shown in the bar graphs on the right. Principal component analysis (**C**) and hierarchical clustering (**D**) of tumor and blood samples based on RNA-seq profile of CD4+ T cells from tumor (blue) and blood (red). (**E**) Pearson correlation of gene expression and differential methylation (differential methylation sites within 10 kb upstream and 100 bp downstream of the nearest gene); red vertical lines indicate positive (right corner) and negative (left corner) correlation beyond 0.7.

In order to associate the unique DNA methylation pattern observed in GBM infiltrating CD4+ T cells with its transcript profile, we performed RNA sequencing (RNAseq) analysis of CD4+ T-cells isolated from both tumor and blood samples of same five GBM patients (Figure 1A). The RNA transcriptome revealed substantial differences between CD4+ T cells from tumor and blood of GBM patients in their RNA expression pattern: 341 genes with log2 fold change of 2 or more and *p* value < 0.01 were shown to be dysregulated in these cells in GBM relative to their expression in blood. Of the 341 genes, compared to CD4+ T-cells from the blood, 191 (56%) genes were downregulated and 150 (44%) genes upregulated, in tumor infiltrating CD4+ T cells (Figure 2A). Among the top 10 upregulated genes in tumor infiltrating CD4+ T cells was *SPP1*, the gene encoding Osteopontin (aka early T lymphocyte activation 1), that binds to integrin receptors on T-cells for cell adhesion and migration, and also responsible for enhancing production of IFNγ and IL12 while reducing IL10, implying that GBM induces CD4+ T-cell migration to the TME (Figure 2B). Principal Component Analysis (PCA) and unsupervised Hierarchical Cluster Analysis (HCA) of DEGs showed individual variability between blood and tumor samples from the patients (Figure 2C and 2D). In addition, HCA of DEG showed similar grouping as DMR, suggesting gene expression and DNA methylation patterns are interconnected. Pearson correlation analysis performed by integrating DMR and DEG datasets showed slight negative linear relationship between gene expression and DMR methylation (median –0.03). The number of differential sites in the negative region (–2.5 to –1.0, to the left of the vertical blue line) was more compared to the positive region (2.5 to 1.0, to the right of the vertical blue line). Furthermore, the regions representing strongly negative and positive linear relationship of differential sites (–1.0 to –0.8 and 0.8 to 1.0, respectively, shown in red color bars in Figure 2E) was moderately more towards the negative region. Collectively the data shows that in tumor infiltrating CD4+ T-cells, DNA methylation might regulate certain gene expression, where hypomethylation of a DMR corresponds with the expression of the corresponding gene and vice versa. Such correlation has been reported in CD4+ T-cell subsets [25], however we for the first time show this in the context of GBM infiltrating CD4+ T cell compartment.

### CD4+ T-cell lineage specific epigenetic signature in GBM

**Figure 3:**
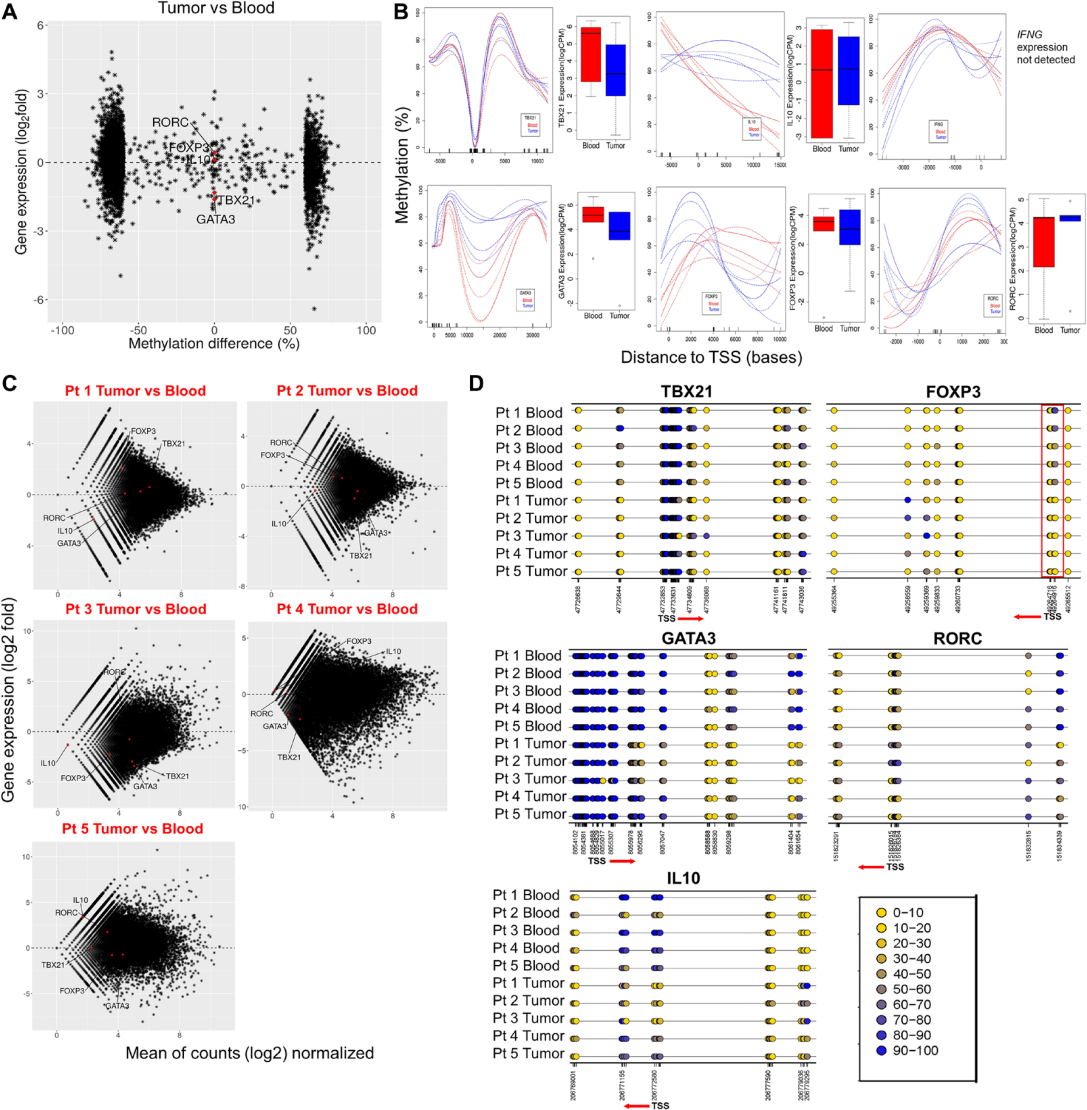
Methylation signature and gene expression alterations of CD4+ lineage specific genes in tumor CD4+ T cells vs blood. (**A**) Methylation difference (%) around promotor regions (horizontal axis) plotted against expression of the corresponding genes (vertical axis; log2 values from RNA-seq) in CD4+ T cells. Select CD4+ T cell lineage genes including TBX21 (Th1), GATA3 (Th2), RORC (Th17), iTreg (FOXP3) and IL10 are marked as red dots. (**B**) Methylation profiles across select genes presented as methylation difference (%, vertical axis). Horizontal axes denote distance from the TSS in bases. On the right side of each methylation plot, the presented boxplots represent the expression (log CPM values from RNA-seq analysis) of the corresponding genes in blood (red) and tumor (blue) CD4+ T cells. (**C**) Transcript profiles of individual tumor CD4+ T cell samples normalized to all blood samples combined is shown. Vertical axis represents gene expression as log2 fold. The red labels inside the plot represents each tumor sample names compared to blood. Each red dot inside the plots represent the select genes of CD4+ T cell lineage included for the analysis. Genes above and below the horizontal line (marked as 0 in the center of the plots) represents up and downregulated genes, respectively. (**D**) Detailed methylation differences in the CpG islands within selected lineage specific genes and their TSS are presented as color coded rings (yellow (0) being hypomethylated and blue (100) being hypermethylated). Below each plot, the numbers indicate genomic position of individual CpG sites. The red arrow and its direction indicate TSS and the direction of transcription of the gene.

Different T cell population has been shown to have specific regions in the genes with differential methylation pattern [26–29]. One of the best examples of this is the identification of a Treg cell–specific hypomethylated region located in the first intron, called ‘conserved non-coding sequence 2’ (CNS2) of Foxp3 [30, 31]. This study was extended further into lymphatic organs and identified a Treg cell specific CpG-hypomethylated pattern in the thymic Treg population [32]. Since primary role of CD4+ T-cells is to orchestrate a lineage specific immune response against pathological antigens, we looked for specific CD4+ T cell sub-population signatures in the tumor infiltrating CD4+ T cells. Upon plotting the mean methylation difference (tumor infiltrating CD4+ T cells – blood CD4+ T cells) of DMRs located in promotors and intragenic regions against RNA-expression data of the corresponding genes, we found moderate to distinct anti-correlation for the association of DNA methylation with gene expression (Figure 3A). We specifically looked for DNA methylation and RNA expression correlation for four major CD4+ T-cell lineage specific genes: TBX21 (Th1), GATA3 (Th-2), FOXP3 (iTreg) and RORC (Th17) as well as the major immunosuppressive (IL10) and pro-inflammatory (IFNγ) genes. We observed that there is very high individual variability in DNA methylation pattern of these genes (promotor and intragenic regions) in GBM tumor infiltrating CD4+ T cells (Figure 3B). However, TBX21, GATA3 and IL10 showed visibly appreciable relationship between DNA methylation and RNA expression. With respect to FOXP3 expression, we observed an increased methylation in the gene body, after the promotor region, and increased RNA levels in CD4+ T cells from tumor. In line with this, as shown in Supplementary Figure 1A, we found significant increase in the percentage of FOXP3 positive cells in tumor compared to blood CD4+ T cells. These data suggest that distinct DNA methylation patterns in tumor infiltrating CD4+ T cells is a unique epigenetic signature of these cells in GBM patients.

Whole genome bisulfite sequencing (WGBS) provides resolution at the level of a single CpG. Thus, we used this to study nucleotide level identification of DNA methylation for genes specific to CD4+ T cell subpopulations. In order to further explore significant individual variation seen in the overall DNA and RNA data, we plotted the RNA expression data of each individual patient’s tumor infiltrating CD4+ T cell samples against blood (Figure 3C), and observed that expression of most of the lineage determining genes as well as the major cytokines showed variability among patients, except for FOXP3, which was upregulated in most patients (3 of 5 patients). Exploration of DNA methylation status of these genes at the individual CpG levels (Figure 3D), also showed high individual variability for TBX21, GATA3, IL10 and RORC, indicating possible involvement of other mechanism for gene regulation besides DNA methylation. However, DNA methylation status on FOXP3 distinctly correlated well with the RNA levels. The CpG in the promotor region of FOXP3 of all the tumor samples were hypomethylated (highlighted as red box, Figure 3D, lower right panel) while there was increased methylation in the gene body. Thus, implying that DNA methylation of FOXP3 promotor and gene body is unique in GBM infiltrating CD4+ T cells.

### Epigenetic landscape of GBM infiltrating CD4+ T cells

**Figure 4:**
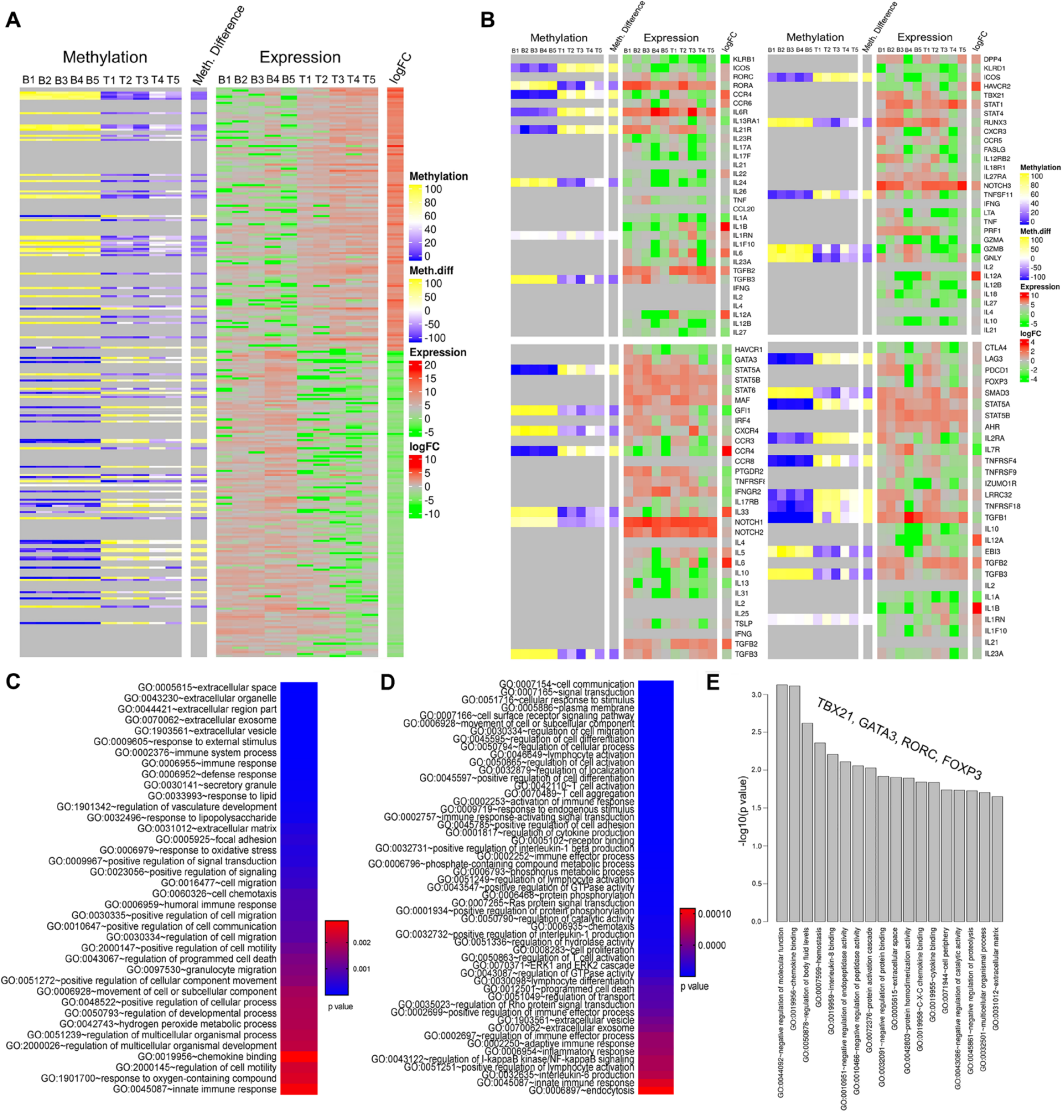
Epigenetic landscape of tumor infiltrating CD4+ T cells: focus on genes related to specific subpopulations. (**A**) Heatmap showing methylation status and expression profiles of the corresponding genes in individual patient’s blood (5 samples on the left-hand side) and tumor CD4+ T cells (5 samples on the right-hand side). The labels on top of the heatmap represents DNA methylation (left) and RNA expression (right) levels. In the methylation data, yellow bands indicate hyper and blue represents hypomethylation. Similarly, green is for down and red for upregulated genes in the expression data. (**B**) Heatmap showing methylation difference and the gene expression level of set of genes corresponding to four predominant CD4+ T-cell lineage: Th17 cells (top left panel), Th1 (top right panel), Th2 (bottom left panel) and iTreg (bottom right panel). The color codes are same as described for panel A. (**C**–**E**) represents top biological pathways obtained from gene ontology analysis using genes associated with the DMRs, dysregulated genes from RNA-seq analysis and gene set comprising specific CD4+ T cells, respectively. Color keys in panels C and D represent *p* values where blue being the lowest *p* value.

Since we noted significant inter patient variability in the DNA methylation and RNA expression of lineage determining genes, we wanted to delineate the effect of GBM mediated epigenetic modification of these transcription factors on the set of downstream genes belonging to the four major lineage types. First, with our integrated parallel analysis of DNA methylation and RNA expression data we observed that overall RNA expression pattern correlated well with DNA methylation status (Figure 4A). There was a visible difference in the DNA methylation pattern in tumor infiltrating CD4+ T cells compared to blood and the corresponding RNA expression values reflected the effect of DNA methylation pattern. When we investigated the relation between DNA methylation and RNA expression of the set of genes specific to CD4+ T-cell lineage specific subpopulations (Figure 4B) we observed that several genes’ expression pattern correlated with the DNA methylation pattern in GBM patients, however, there were others that did not, again implying that DNA modification is not the only mechanism for gene expression changes in tumor infiltrating CD4+ T cells. After scanning through all the genes in the sets for each T cell lineage we observed that, for example, in the Th17 specific gene set (Figure 4B, top left panel), IL1RN was upregulated and its DNA methylation indicated hypomethylation. On the other hand, IL6R and IL21R had hypermethylation and the expression level showed downregulation. In the Th1 related gene set (Figure 4B, top right panel), only TNSF11 showed good correlation where its expression was down and the DNA methylation was higher in CD4+ T cells from tumor. With regards to Th2 related gene set (Figure 4B, lower left panel), IL33 and NOTCH1 were up and STAT5A was down while correlating well with their DNA methylation. In particular, T reg lineage cell gene set (lower right panel in Figure 4B) had maximum number of genes which had expression pattern correlating really well with DNA methylation pattern. SMAD3 and IL1RN were up while IL2RA and TGFB1 were down in iTreg related gene set and the DNA methylation also corroborated well. As expected, we observed several genes that had expression level independent of DNA methylation in all these gene sets indicative of other mechanisms regulating these genes.

### Gene-Ontology pathway analysis

In order to understand what biological pathways these genes regulated, we performed gene ontology analysis by separately taking genes associated only to either the DMRs, DEGs or CD4+ T cell specific lineages. As expected, the DMR associated genes are involved in various T cell biological functions like T cell activation, aggregation, activation of immune response, positive regulation of interleukin-1 beta production, immune effector process, signal transduction, programmed cell death, chemotaxis, interleukin 6 production, endocytosis etc., all of which were in the lowest side of *p* value (Figure 4C). Pathways associated with the differentially expressed genes (Figure 4D) were also majorly related to immune system functioning and cell migration. Furthermore, we observed that majority of the pathways which were associated with the DMR genes were also associated with the DEGs suggesting that the genes differentially expressed in tumor CD 4+ T cells may be associated with differential methylation. Gene ontology enrichment analysis on the gene set comprising of TBX21, GATA3, RORC, FOXP3 showed diverse gene ontologies (Figure 4E). The top pathway in this analysis was “negative regulation of molecular function”. The observed alterations of these pathways in tumor associated CD4+ T cells suggested the functional significance of these pathways in the CD4+ T cells differentiation and function.

### Ligands for some of the differentially expressed receptors on tumor infiltrating CD4+ T-cells were upregulated on the GBM tumor cells

**Figure 5:**
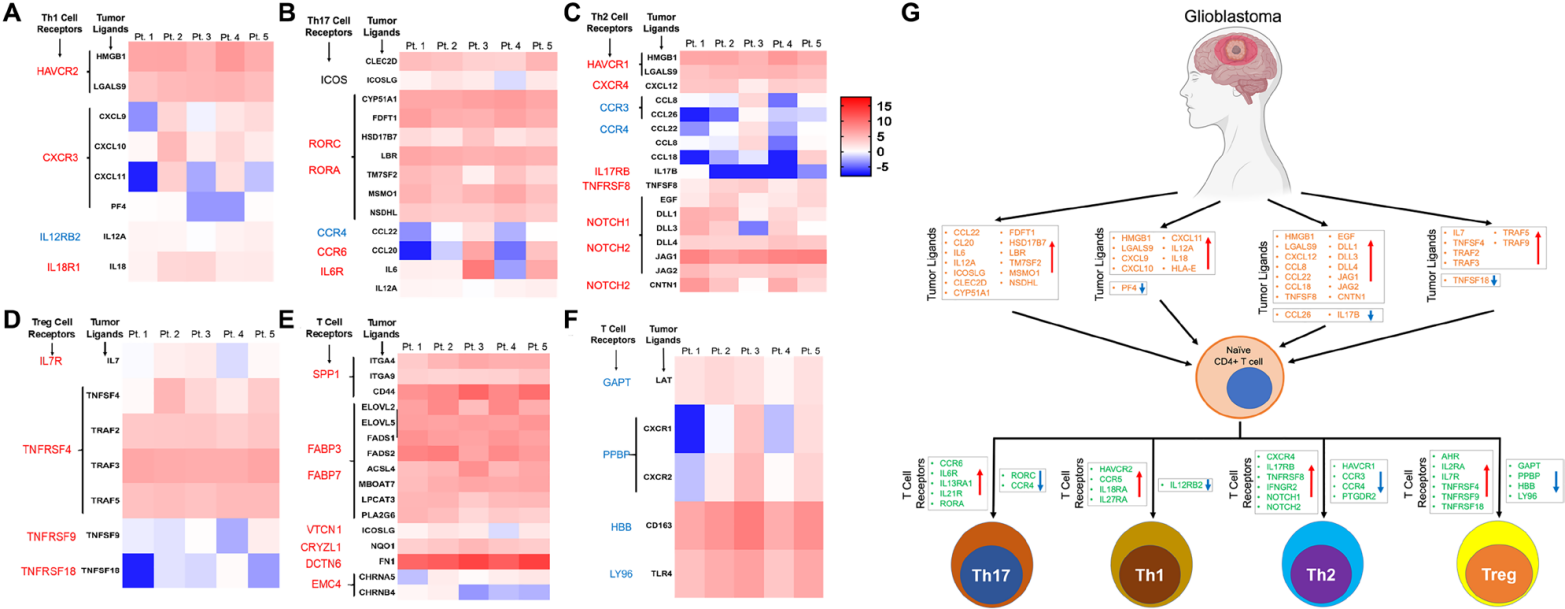
Expression pattern of ligands of corresponding receptors specific for CD4+ T cell lineages in GBM tumor tissue. (**A**–**D**) The heatmaps show expression of the ligands in tumor tissue and graphical representation of their corresponding receptor expression level on the CD4+ T-cells in the four major subtypes: Th1 (panel A), Th17 (panel B), Th2 (panel C) and iTreg (Panel D) CD4+ T cells in GBM tumors. Font colors (red; upregulated and blue; downregulated) of the receptors on the left to the heatmap represent expression pattern. The heatmap color keys indicate up (red) and down (blue) regulation of a gene. Expression of genes only in tumor CD4+ T cells are shown. Each patient is labelled as Pt followed by a number (on top of the heatmap). (**E** and **F**) represents top up and down regulated, respectively, ligands or receptors in CD4+ T cells from tumors. The color keys of the heatmap is similar to one described earlier for panels A–D. (**G**) Graphical representation of our findings. GBM differentially expresses various immune modulating ligands (up and down regulation showed as up and down arrows) which in turn bind to specific receptors on undifferentiated naïve CD4+ T cells. This results in modulation of the signaling pathways leading to the lineage specific Th1, Th17, Th2, and iTreg cells. All the dysregulated ligands and receptors were combined in this graphical abstract to show their expression patterns in respective tumor infiltrating CD4+ T cell lineages. Orange color genes are ligands and one in blue are their receptors. Direction of the arrows in the box indicates expression level of the genes (upward arrow; upregulated and vice versa).

Since tumor infiltrating CD4+ T cells had distinct epigenetic signature compared to the CD4+ T cells from the blood in the same patient, we hypothesized that TME induces these changes in tumor infiltrating T-cells. Thus, using RNA sequencing of the tumor cells from the same patients we explored the expression level of various ligands and cytokines that alters corresponding receptors on the CD4+ T-cells. Interaction between ligand and its specific receptor is the first step in bringing about the functional change in immune cells function including CD4+ T cells. For this interaction to occur, the expression of both the ligands and its receptors are equally important. Thus, we identified various receptors that were upregulated in each lineage specific CD4+ subpopulation and then analyzed their ligand expression status in on the tumor cells (Figure 5A–5D). Interestingly we found that several ligands and its receptors showed positive correlation pattern: if ligands were upregulated in the tumor cells, receptors were also upregulated (upward pointing red arrow) on the lineage specific CD4+ T-cells and vice versa (Figure 5A–5D). For example, in Th1 lineage, the receptor HAVCR2 is upregulated and its ligands (HMGB1, LGALS9) are also upregulated (Figure 5A), however although CXCR3 is upregulated on Th1 T-cells not all its ligands are up-regulated and they show inter-patient variability. In the Th17 lineage (Figure 5B), RORC (receptor) is upregulated on the tumor infiltrating CD4+ T-cells and its ligands (CYP51A1, FDFT1, HSD17B7, LBR, TM7SF2, MSMO1, NSDHL) are also upregulated on the tumor cells, implying GBM expresses ligands that induces Th17 phenotype in tumor infiltrating helper T-cell. A trend similar to that seen in Th1 was seen in case of Th2 group also (Figure 5C). Interestingly, the most striking correlation was observed in case of iTreg panel where there were clear matching expression patterns for both up and downregulated receptor/ligand sets (Figure 5D). For example, TNFRSF4 (receptor) is upregulated and all the corresponding ligands (TNFSF4, TRAF2, TRAF3, TRAF5) were also upregulated, while TNFRSF9 was down and its ligand TNFSF9 was also down. Next, we looked at the top dysregulated ligand/receptor pairs in tumor infiltrating CD4+ T cells and tumor. Best matching ligand/receptor sets in terms of their expression was observed for receptors SPP1, FABP3, FABP4, VTCN1, CRYZL1 and DCTN6, which were all upregulated, and their ligands were also upregulated in all the patients (Figure 5E). However, in the top downregulated ligand/receptor sets (Figure 5F), we did not see a very consistent pattern like we did in top upregulated gene sets. Overall, this analysis provided evidence that GBM expresses certain ligands and it modulates the lineage of tumor infiltrating T-helper cell through their corresponding receptors and modulate the anti-tumor immune response in the TME (Figure 5G).

## DISCUSSION

Our study for the first time, reports that GBM induces diverse endogenous immune response and there is very significant inter-patient variability on the type of predominant T helper immune response in the TME. There is a distinct methylation signature of the tumor infiltrating CD4+ T cells compared to CD4+ T cells from blood from the same patient. This implies that GBM TME might influence the tumor infiltrating CD4+ T cells by way of extensive epigenetic reprogramming thus modulating the endogenous anti-tumor immune response. Since, DNA methylation is involved in silencing (or releasing gene suppression upon demethylation of promotor regions) gene expression at the transcription level [33], one would assume that GBM regulate the expression of the genes in tumor infiltrating CD4+ T cells by modulating their DNA methylation. As it is evidenced that several genes (including TP53, BRCA1 etc.) are regulated by DNA methylation in other cancers [34–37] we also believe altering the DNA methylation in the tumor infiltrating CD4+ T cells, especially the key lineage specific transcription factors and key cytokines is an inherent characteristic of GBM to regulate effector phenotypes in these cells.

Our finding is in line with growing body of evidence that suggests that epigenetic modifications set thresholds of gene expression that determine T cell fate and function [11]. In addition, epigenetic modifications have been identified as a possible mechanism by which the local microenvironment establishes the tissue-resident characteristics in macrophages and Tregs [25, 38]. Similar mechanisms could be important for shaping the identity of tumor infiltrating CD4+ T cells within the TME. This phenomenon of TME induced epigenetic changes in immune cells has been observed in other cancer model [28, 39]. Thus, based on these reports and our present findings, we believe that GBM dictates the fate of tumor infiltrating CD4+ T cells by altering the DNA methylation of key genes that determine the cell’s fate.

Previous studies with normal healthy donor human primary CD4+ T cells showed that DNA methylation in gene promoter region is not always a repressive mark, where up to 27% of methylated genes are actively expressed in naïve CD4+ T cells and the distance of methylation site from the TSS determine gene repression [40]. Our data show that majority of the DMR’s in tumor infiltrating CD4+ T cells were concentrated between ~2Kb upstream and downstream to the TSS, implying that tumor influences epigenetic changes in tumor infiltrating helper T-cells. In agreement with published literature our data also show that out of a cluster of genes belonging to specific lineage, not all genes are regulated by repressive methylation, there were many genes that showed pattern of permissive DNA methylation, implying other mechanisms of gene regulation is also at play in the TME besides epigenetic modification.

In addition, biological pathways analysis has shown that in human CD4+ T-cells transcriptionally repressive DNA methylation peaks were associated with genes involved in immune response and T cell differentiation, whereas transcriptionally permissive DNA methylation peaks were associated with genes involved in cell growth, proliferation and cell signaling [40]. In line with this observation, our data on hyper- and hypomethylation pattern of specific locations on key CD4+ lineage specific differentiation genes TBX21, GATA3, RORC and FOXP3, along with the observed differential gene expression, suggests an association between tumor influenced methylation status and the expression patterns of these genes in tumor infiltrating CD4+ helper T cell. In addition, we further observed that several genes dysregulated in the CD4+ T cells from GBM were related to immune cell functioning. For example, *SPP1* encoding osteopontin (OPN) and VTCN1 (aka B7-H4) are the genes known to be involved in cytokine production and negative regulation of T cell mediated immunity [41], suggesting a deficiency in T cell-mediated immunity in tumor associated CD4+ T cells. Furthermore, OPN function is also involved in CD8+ T cells’ migration, activation and viability [42] and B7-H4 functions as a negative regulator of T cell responses, inhibiting T cell proliferation, IL-2 production, and cell cycle [43, 44]. GBM is known for its heterogeneity and exhibit significant intra-tumoral and inter-patient variation in expression of various antigens and ligands [45, 46]. We also found significant variability in the gene expression of various ligands on GBM cells between our patient population. Furthermore, our data show striking inter patient variability in the epigenetic signature and key lineage specific CD4+ T cell transcription factor methylation status of tumor infiltrating CD4+ T-cell but not the CD4+ T cells from the blood. Taken together our findings implicate a possible critical role played by the GBM microenvironment in shaping anti-GBM T-helper cell response by the means of epigenetic regulation in the TME.

Ligands and their receptors on T cells interact to induce a signaling pathway during various stages including activation and differentiation of the CD4+ T cells from its naïve stage to a specific subpopulation of effector T cell [47]. We observed paired expression of several ligands on the GBM cells and its receptors in specific T cell subpopulations. These observations implied that tumor cells in the GBM microenvironment expressing specific ligands influence tumor infiltrating CD4+ T-cells by interacting with the corresponding receptors on the T-cells. In characteristic with GBM’s behavior inter-patient variability was also reflected in the pattern of ligand receptor interaction. Some of the important ligands and their respective receptors, for example CCL20 and CCR6, CXCL11 and CXCR3, IL17B and IL17RB, TNFSF18 and TNFRSF18 (iTregs) etc., had opposite expression pattern in certain patients while there was same expression pattern in others. It is reported that interaction between CCL20 and CCR6 is important for effector T cell chemotaxis [48, 49], CXCL11 and CXCR3 is chemotactic for activated T cells [50], IL17B stimulates release of IL1 [51] and TNFSF18-TNFRSF18 interaction is required for T cell responses [52]. GBM TME might disrupt the expression of these ligand receptor pairs, thus disrupting important immune functioning signaling pathways favoring cancer cell survival. This also provides an explanation why certain patients respond better to immunotherapy than others in therapeutic clinical trial setting [53].

In summary, in the present clinical corelative report, we demonstrated that differential DNA methylation pattern might influence gene expression in tumor infiltering CD4+ T cells as compared to circulating blood CD4+ T cells in GBM patients. Our findings provide evidence that GBM might be influencing the state of tumor infiltrating CD4+ T cells by epigenetic modification in the form of DNA methylation of key immune function regulating genes and influencing the fate of helper T cells in the GBM TME. Based on our observations we believe that perhaps epigenetic interaction between GBM and tumor infiltrating CD4+ T cells is responsible for the immunosuppressed state seen in the GBM patients. Our data convincingly show that there is significant inter-patient variability in the GBM tumor ligand expression of various T-cell modulating ligands and consequently striking differences in the methylation pattern and gene expression in tumor infiltrating CD4+ T-cells. This has a very strong implication for selecting future patients for immunotherapy trials who will have better likelihood of responding to immunotherapy than others based on their tumor immune signature. The findings from our corelative study needs to be further validated in the experimental setting.

## MATERIALS AND METHODS

### Patient samples

All patients included in this study were newly diagnosed with GBM. We obtained written consent from each patient to participate in this study. The study protocol was approved by the IRB of Indiana University. Tumor tissue and matched blood was collected during surgery after intra-operative diagnosis of GBM was made. Final pathology and molecular subtype were confirmed by clinical neuro-pathologist. Five GBM patients’ matching blood and tumor were used for the study. None of the patients had undergone any treatment for GBM prior to surgical resection for sample collection. All the patients included in this study were steroid naïve. Clinical characteristics of the patients are described in Table 1.

### Isolation of cells from blood and brain

Total leukocytes from blood and lymphocytes from tumor tissues were isolated. To lyse red blood cells (RBCs) from blood, 2 mL of blood was mixed with 8 mL of ACK lysing buffer (cat# 118-156-101, Quality Biologicals, MD, USA), mixed well and centrifuged at 1200 rpm for 5 min at room temperature (RT). Cell pellets were washed once with DPBS and later used for sorting of CD4+ T cells. Tumor tissues from GBM patients were obtained during surgical resection and processed right after harvesting. As much as possible, samples were processed under sterile condition and minimal RNAse contamination. To isolate lymphocytes, tissues were first homogenized in a 70 μ cell strainer using a syringe plunger and in 2% FBS containing RPMI medium. Cells were pelleted by centrifugation at 1500 rpm for 5 minutes at 4°C. Optionally, if present, RBCs were lysed using 5 ml of ACK Lysing Buffer, incubating for 5 min at RT, centrifuging at 1500 rpm for 5 minutes and washing with DPBS. Cell pellet is then resuspended in 8 mL of 30% Percoll medium and layered over 4 mL of 70% Percoll layer. The Percoll gradient column is centrifuged at 500g (~1500 rpm) for 30 min at 4°C in a swing out bucket centrifuge without brake. Lymphocyte ring above the 70% Percoll layer is gently pipetted and washed with DPBS by centrifugation at 1500 rpm for 5 min and resuspended in 2% FBS containing RPMI. These lymphocytes were used for sorting od CD4+ T-cells.

### Sorting of CD4+ T cells

Single cell suspension of the lymphocytes obtained from blood and tumors were taken for isolation of CD4+ T lymphocytes using the CD4 multiSort Kit using the protocol described by manufacturer (MACS MiltenyiBiotec, Auburn, CA, USA). Briefly, cells were incubated with anti-CD4 multiSort MicroBeads (20 μl/10^7^ cells) in the refrigerator for 15 min, washed with 2 ml buffer (provided in the kit) and re-suspended in 500 μl of buffer. Magnetic separation of the CD4 labeled cells was performed by passing the cells through the MS column. Columns were washed 3 times and the CD4 labelled cells were eluted with the buffer. The collected cells were washed and proceeded for isolation of DNA and RNA.

### Purification of DNA and RNA

DNA was isolated from the purified CD4+ T cells using the DNAeasy kit as per the manufacturer instructions (Qiagen Inc, CA, USA). Briefly, cells were lysed with lysis buffer, applied the lysate into a DNA purification column, washed as per kit instruction after proteinase K treatment and eluted with 30–50 μl of elution buffer, followed by Nanodrop spectrophotometer quantification. Total RNA from CD4+ T cells was isolated using the RNeasy Plus Mini kit following the procedure described by the manufacturer (Qiagen, CA, USA). Briefly, CD4+ T cells were lysed using the RLT Plus buffer, transferred the lysate into a genomic DNA removal column and took only the flow through for use in RNA isolation. Rest of the procedure were as described by the kit manufacturer. The RNA was eluted in 30–50 μl of RNAse free water, quantified and stored at –80ºC until further use. Tumor total RNA was isolated from the tumor tissue after removal of the lymphocytes using the RNeasy Plus Mini kit following the manufacturer’s instructions (Qiagen Inc, CA, USA) without any modification. After the final wash, the bound total RNA was eluted from the columns using 30 μl of RNase-free water and determined the quantity and purity of the RNA using the Nanodrop spectrophotometer and stored at –80ºC until further use.

### Whole genome bisulfite sequencing

We employed Illumina TruSeq Methyl Capture EPIC method for human gDNA sequencing. To do this, human genomic DNA was first evaluated for its quantity and quality using Agilent TapeStation 4200 and Thermo Fisher Qubit 3.0. Five hundred nanograms of high quality gDNA were used for library preparation. The protocol followed was as instructed by the kit manufacturer. DNA library preparation first included fragmentation by Covaris S2 to average size range of 150-200bp, end-repair, 3’ A-tailing, and adaptor ligation. Libraries were then pooled in groups of 4 libraries in equal aliquots, followed by 2 rounds of hybridization and capture using Illumina optimized EPIC probe set covering > 3.3 million targeted CpG islands and CpG sites (hg19), bisulfite conversion, and amplification (provided in Illumina TruSeq Methyl Capture EPIC Library Prep Reference Guide, Document # 1000000001643 v01, May2017). Each resulting captured library pool was quantified and its quality accessed by Qubit and Agilent Bioanalyzer, and multiple library pools were further combined in equal molarity. Five microliters of 3 nM pooled libraries for each lane were then denatured, neutralized and applied to the cBot for flow cell deposition and cluster amplification, before loading on to HiSeq 4000 for 100b paired-end sequencing (Illumina, Inc.). Five percent PhiX DNA was added to each library pool during cluster amplification to boost diversity of the library. Each flow cell has 8 lanes and each lane generates approximately 300-350 million reads.

### RNA sequencing and gene expression analysis

We utilized Clontech’s SMARTer RNA Pico Kit v2 for library preparation and used Illumina platform for sequencing. The protocol followed was as instructed by the kit manufacturer. Total RNA was first evaluated for its quantity and quality using Agilent Bioanalyzer 2100. RIN (RNA Integrity Number) and DV200 (% total RNA above 200b) were obtained to decide RNA fragmentation time. Depending on the integrity and DV200 of RNA, the fragmentation time ranges from 4 minutes down to 1.5 minutes with longer time for higher quality RNA. No fragmentation would be conducted for FFPE, or RNA samples of DV200 < 50%. Two hundred fifty picograms to ten nanograms of total RNA per sample were used for library preparation. Library preparation included fragmentation, first-strand cDNA synthesis, index adaptor ligation, rRNA depletion, and library amplification by PCR, following the standard protocol of Clontech SMARTer RNA Pico Kit V2 (Takara Clontech Laboratories, Inc.). The resulting library was quantified and its quality accessed by Qubit and Agilent Bioanalyzer, and multiple libraries were pooled in equal molarity. Average size of library insert was about 150–200b. Five microliters of 2 nM pooled libraries per lane were then denatured, neutralized and applied to the cBot for flow cell deposition and cluster amplification, before loading on to HiSeq 4000 for 75b paired-end sequencing (Illumina Inc., CA, USA). Each lane generated approximately 300–350 million reads. A Phred quality score (Q score) was used to measure the quality of sequencing. More than 90% of the sequencing reads reached Q30 (99.9% base call accuracy).

For tumor RNA sequencing, we have used KAPA RNA HyperPrep methods for total RNA sequencing. Total RNA was first evaluated for its quantity, and quality, using Agilent Bioanalyzer 2100. One hundred nanograms of total RNA were used. Ribosomal RNA was removed from total RNA using standard protocol of the KAPA RNA HyperPrep Kit with RiboErase (HRM) Globin (Roche Corporate, USA, Catalog #KK8562). After the depletion of rRNA, cDNA library preparation was carried out including RNA fragmentation, cDNA synthesis, ligation of index adaptors, and amplification, following the KAPA RNA Hyper Prep Kit Technical Data Sheet, KR1520 – v2.17 (Roche Corporate). Each resulting indexed library was quantified and its quality accessed by Qubit and Agilent Bioanalyzer, and multiple libraries were pooled in equal molarity. The library pool was then sequenced in 75b single read format on NextSeq 500 (Illumina Inc., CA, USA). More than 500 million reads were generated and 91% of the sequencing reads reached Q30 (99.9% base call accuracy). A Phred quality score (Q score) was used to measure the quality of sequencing.

### Bioinformatics data analysis

For DNA methylation sequencing data, sequences were aligned using Bismark v0.18.2 with the Bowtie2 option turned on. Coverage2cytosine in Bismark was used to generate cytosine methylation report which was then fed into methyl Kit v1.4.1 [54]. Bases with too low (< 10×) or too high coverage (bases that had more than the 99.9th percentile of coverage in each sample) were discarded. Differentially methylated sites were calculated using a minimum *q*-value of 0.01 and methylation difference of 60%. Differential methylated sites overlapping with promoter (upstream 10000bp and downstream 100bp of transcription start site) of genes were associated with those genes. Circos plots were generated for comparative analysis [55].

For RNA-Seq data, the reads were mapped to the human genome using STAR (v2.5) RNA-seq aligner with the following parameter: “--outSAMmapqUnique 60” [56]. Uniquely mapped sequencing reads were assigned to Gencode 25 genes using featureCounts (v1.6.2) with the following parameters: “-s 2 –p –Q 10” [57]. The data was filtered using read count per million (CPM) > 0.5 in more than 2 of the samples, normalized using TMM (trimmed mean of M values) method and subjected to differential expression analysis using edgeR (v3.20.8) [58, 59].

## SUPPLEMENTARY MATERIALS




