# Supplementary material for: Genome wide DNA methylation landscape reveals glioblastoma’s influence on epigenetic changes in tumor infiltrating CD4+ T cells
Source: Oncotarget. 2021 May 11;12(10):967–81. doi: 10.18632/oncotarget.27955 (PMC8121608; doi:10.18632/oncotarget.27955)
Supplement: Supplementary file 1 [file oncotarget-12-967-s001.pdf]

Genome wide DNA methylation landscape reveals glioblastoma's influence on epigenetic changes in tumor infiltrating CD4+ T cells

SUPPLEMENTARY MATERIALS

A

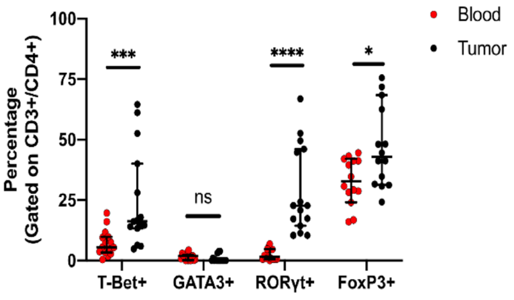

B

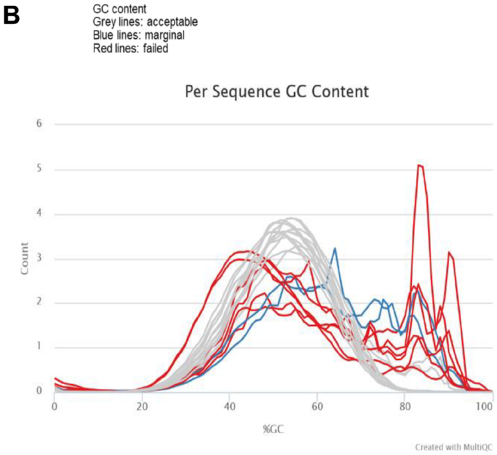

C

Mapping Statistics

	Pt_10_Blood	Pt_10_Tumor	Pt_12_Blood	Pt_12_Tumor	Pt_13_Blood	Pt_13_Tumor	Pt_8_Blood	Pt_8_Tumor	Pt_9_Blood	Pt_9_Tumor
Raw reads	32,875,234	31,965,901	35,777,966	39,999,860	35,382,843	32,584,937	38,389,674	31,471,844	33,149,386	34,742,111
Mapped reads	11,671,333	12,459,808	14,226,717	22,297,971	15,205,408	26,800,922	14,283,486	14,635,059	11,953,270	12,079,359
High quality reads	10,729,765	11,689,280	13,311,118	21,157,713	14,284,822	26,407,504	13,367,376	13,807,697	10,965,133	11,153,847
Mapped to gene	679,483	1,593,489	2,342,223	9,943,621	1,613,553	2,102,247	811,500	657,595	687,016	724,492
coding	217,046	221,406	82,843	141,519	137,430	218,272	302,346	218,572	208,768	245,257
coding-rev	232,234	595,359	748,736	4,125,451	363,736	776,781	308,236	232,770	238,077	254,407
intergenic	5,012,275	4,896,920	2,748,197	3,882,942	4,695,720	13,200,727	6,437,716	6,995,269	5,092,376	5,332,952
intron	2,207,456	2,061,279	982,555	1,176,639	1,796,242	4,163,824	2,844,597	2,936,924	2,199,541	2,396,452
intron-rev	2,145,779	2,233,537	2,051,526	5,355,184	2,260,536	6,304,074	2,741,449	2,793,616	2,146,628	2,297,822
junction	10,559	252,875	497,705	2,579,368	186,227	388,239	3,903	11,202	19,242	7,476
mitochondrial	335,179	562,664	5,005,507	880,406	3,618,963	252,822	944	9,011	478,957	14,505
other-exon	28,040	31,771	35,689	65,045	55,269	56,774	35,148	31,831	28,922	30,411
other-exon-rev	50,305	166,502	528,933	1,626,524	598,435	170,410	36,956	34,573	39,380	32,642
promoter	132,799	137,764	110,801	119,435	126,748	190,772	173,696	147,684	140,177	144,966
promoter-rev	121,927	166,940	124,383	174,306	132,218	193,071	168,496	137,986	124,847	135,743
coding%	2.02	1.89	0.62	0.67	0.96	0.83	2.26	1.58	1.90	2.20
coding-rev%	2.16	5.09	5.62	19.50	2.55	2.94	2.31	1.69	2.17	2.28
intergenic%	46.71	41.89	20.65	18.35	32.87	49.99	48.16	50.66	46.44	47.81
intron%	20.57	17.63	7.38	5.56	12.57	15.77	21.28	21.27	20.06	21.49
intron-rev%	20.00	19.11	15.41	25.31	15.82	23.87	20.51	20.23	19.58	20.60
junction%	0.10	2.16	3.74	12.19	1.30	1.47	0.03	0.08	0.18	0.07
mitochondrial%	3.12	4.81	37.60	4.16	25.33	0.96	0.01	0.07	4.37	0.13
other-exon%	0.26	0.27	0.27	0.31	0.39	0.21	0.26	0.23	0.26	0.27
other-exon-rev%	0.47	1.42	3.97	7.69	4.19	0.65	0.28	0.25	0.36	0.29
promoter%	1.24	1.18	0.83	0.56	0.89	0.72	1.30	1.07	1.28	1.30
promoter-rev%	1.14	1.43	0.93	0.82	0.93	0.73	1.26	1.00	1.14	1.22
Mapped to gene %	6.33	13.63	17.60	47.00	11.30	7.96	6.07	4.76	6.27	6.50

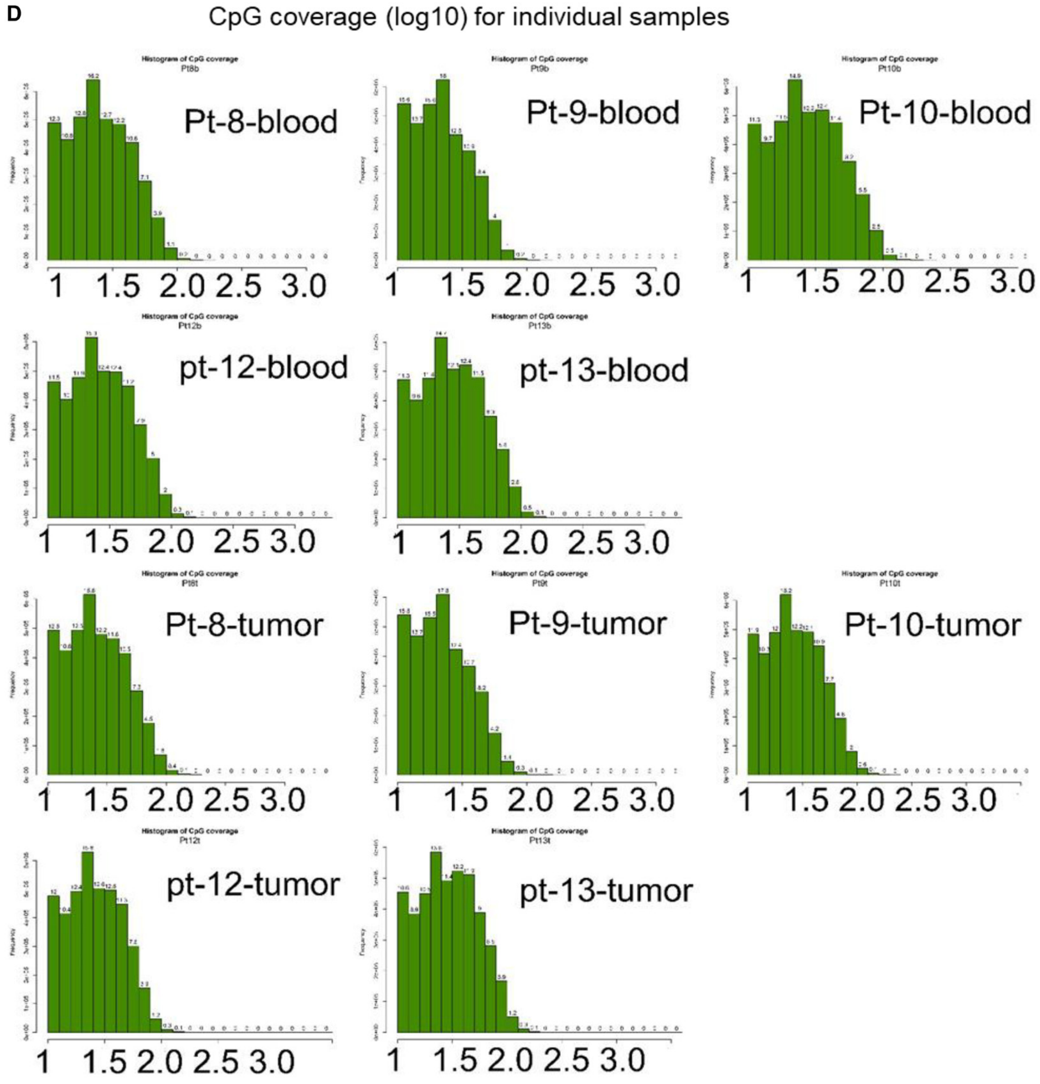

Supplementary Figure 1: (**A**) Flow cytometry data showing percentages of different subpopulations of CD4+ T cells in the GBM tumor infiltrating lymphocytes (black dots) compared to the blood (red dots) from the same individuals. Each dot represents an individual included in the analysis. We performed whole genome bisulfite sequencing of DNA samples from GBM tumor infiltrating CD4+ T cells and also from blood of the same individuals. (**B**–**D**) GC content in the sequencing data, mapping statistics and CpG coverage for each individual, respectively. The data obtained from bisulfite sequencing was analyzed in house in the Department of Bioinformatics, Indiana University School of Medicine, Indiana. The table provides information on mapping stats of all individual samples, hyper and hypomethylated regions in tumor samples etc.

Supplementary Table 1: Top 10 significant DMRs and their associated genes in tumor CD4+ T cells compared to blood CD4+ T cells. See Supplementary Table 1
