# Supplementary material for: Genome wide DNA methylation landscape reveals glioblastoma’s influence on epigenetic changes in tumor infiltrating CD4+ T cells
Source: Oncotarget. 2021 May 11;12(10):967–81. doi: 10.18632/oncotarget.27955 (PMC8121608; doi:10.18632/oncotarget.27955)
Supplement: Supplementary file 2 [file oncotarget-12-967-s002.docx]

**Supplementary Table 1: Top 10 significant DMRs and their associated genes in tumor CD4+ T cells compared to blood CD4+ T cells**

**Hypomethylated DMRs**

| **chr** | **Start** | **End** | **Strand** | **p value** | **q value** | **Meth. diff** | **Gene** | **Distance**  **to TSS** | **Strand** | **Symbol** | **Gene name** |
| --- | --- | --- | --- | --- | --- | --- | --- | --- | --- | --- | --- |
| chr7 | 13985527 | 13985527 | - | 6.80E-171 | 1.09E-167 | -80.6171937 | NM_001163150 | 988 | - | ETV1 | ETS variant 1 |
| chr7 | 1.52E+08 | 1.52E+08 | - | 1.33E-166 | 2.12E-163 | -78.9927911 | NM_022087 | 160175 | + | GALNT11 | polypeptide N-acetylgalactosaminyltransferase 11 |
| chr7 | 1.54E+08 | 1.54E+08 | - | 3.92E-149 | 6.25E-146 | -78.2970783 | NM_001936 | 48064 | + | DPP6 | dipeptidyl peptidase like 6 |
| chr12 | 55720398 | 55720398 | - | 8.66E-149 | 1.38E-145 | -72.7979523 | NM_002905 | 33 | + | RDH5 | retinol dehydrogenase 5 |
| chr12 | 9116198 | 9116198 | - | 2.23E-147 | 3.53E-144 | -79.0086531 | NM_000014 | -237 | - | A2M | alpha-2-macroglobulin |
| chr11 | 67792667 | 67792667 | - | 4.14E-143 | 6.51E-140 | -67.1755801 | NR_024249 | 12670 | - | FAM86C2P | family with sequence similarity 86, member A pseudogene |
| chr7 | 1.54E+08 | 1.54E+08 | - | 5.81E-143 | 9.13E-140 | -78.6157183 | NM_001936 | 48058 | + | DPP6 | dipeptidyl peptidase like 6 |
| chr4 | 1.86E+08 | 1.86E+08 | - | 8.16E-142 | 1.28E-138 | -72.2030142 | NM_001145675 | -69 | - | SORBS2 | sorbin and SH3 domain containing 2 |
| chr16 | 19578675 | 19578675 | - | 5.69E-140 | 8.89E-137 | -73.8967325 | NM_020314 | 23262 | + | C16orf62 | chromosome 16 open reading frame 62 |
| chr2 | 1.82E+08 | 1.82E+08 | - | 7.62E-140 | 1.19E-136 | -70.9836456 | NR_048566 | 135 | + | PPP1R1C | protein phosphatase 1 regulatory inhibitor subunit 1C |

**Hypermethylated DMRs**

| **chr** | **Start** | **End** | **Strand** | **p value** | **q value** | **Meth. diff** | **Gene** | **Distance**  **to TSS** | **Strand** | **Symbol** | **Gene name** |
| --- | --- | --- | --- | --- | --- | --- | --- | --- | --- | --- | --- |
| chr17 | 68296832 | 68296832 | - | 3.74E-147 | 5.92E-144 | 80.11122609 | NM_001267727 | 5339 | + | ARSG | arylsulfatase G |
| chr19 | 55421563 | 55421563 | - | 7.38E-164 | 1.18E-160 | 77.19744024 | NM_014501 | -13607 | - | UBE2S | ubiquitin conjugating enzyme E2 S |
| chr7 | 33041004 | 33041004 | - | 3.42E-154 | 5.46E-151 | 75.32514434 | NM_001166118 | 162 | - | NT5C3A | 5'-nucleotidase, cytosolic IIIA |
| chr7 | 112422365 | 1.12E+08 | - | 1.31E-145 | 2.07E-142 | 74.98984153 | NM_001007245 | -779 | + | IFRD1 | interferon related developmental regulator 1 |
| chr7 | 45027245 | 45027245 | - | 3.57E-181 | 5.74E-178 | 74.50919225 | NM_001029835 | -389 | + | CCM2 | CCM2 scaffolding protein |
| chr7 | 33040885 | 33040885 | - | 7.83E-149 | 1.25E-145 | 73.75721045 | NM_001166118 | 281 | - | NT5C3A | 5'-nucleotidase, cytosolic IIIA |
| chr14 | 100068995 | 1E+08 | - | 3.35E-146 | 5.30E-143 | 71.50550095 | NM_016337 | 3583 | + | EVL | Enah/Vasp-like |
| chr7 | 139107246 | 1.39E+08 | - | 7.35E-148 | 1.17E-144 | 71.35202259 | NM_020119 | 2474 | - | ZC3HAV1 | zinc finger CCCH-type containing, antiviral 1 |
| chr18 | 813497 | 813497 | - | 5.67E-148 | 9.00E-145 | 71.2971586 | NM_005433 | -1172 | - | YES1 | YES proto-oncogene 1, Src family tyrosine kinase |
| chr12 | 54497853 | 54497853 | - | 1.33E-150 | 2.12E-147 | 69.9280807 | NM_005337 | 144 | + | NCKAP1L | NCK associated protein 1 like |
